# Daptomycin-Induced Eosinophilic Pneumonia in a Patient With Persistent Methicillin-Resistant Staphylococcus aureus Bacteremia Undergoing Hemodialysis: A Case Report

**DOI:** 10.7759/cureus.107269

**Published:** 2026-04-17

**Authors:** Bader H Alsawadi, Feras S Beitar, Reenad S Ghunaim

**Affiliations:** 1 Critical Care Medicine, Prince Mohammed Bin Abdulaziz Hospital, National Guard Health Affairs, Al Madinah, SAU; 2 Nephrology, Prince Mohammed Bin Abdulaziz Hospital, National Guard Health Affairs, Al Madinah, SAU; 3 Infectious Diseases, Prince Mohammed Bin Abdulaziz Hospital, National Guard Health Affairs, Al Madinah, SAU

**Keywords:** daptomycin, drug-related side effects and adverse reactions, eosinophilic pneumonia, methicillin-resistant staphylococcus aureus, renal dialysis

## Abstract

Daptomycin-induced eosinophilic pneumonia (DEP), believed to be a T cell-mediated hypersensitivity reaction, is a rare but severe side effect of daptomycin therapy. It manifests similarly to infective pneumonia and commonly affects seriously or critically ill patients. Herein, we describe the case of a 69-year-old man undergoing chronic hemodialysis who received 24 days of daptomycin therapy for recurring methicillin-resistant *Staphylococcus aureus* septicemia and septic arthritis following vancomycin therapy failure. After the patient developed acute hypoxemic respiratory failure, daptomycin therapy was discontinued. Respiratory cultures grew multidrug-resistant Acinetobacter baumannii, initially raising concern for hospital-acquired pneumonia. However, marked eosinophilia (8.6%) and the temporal relationship with daptomycin exposure supported the diagnosis of DEP. Rapid clinical and radiological improvement was observed within 24 hours following daptomycin withdrawal and systemic corticosteroid initiation, although the relative contribution of each intervention is unclear. This report highlights the need for considering DEP in cases of respiratory failure among patients undergoing hemodialysis and receiving daptomycin therapy, even with positive culture findings for respiratory infections.

## Introduction

Daptomycin is an established alternative to vancomycin for the treatment of methicillin-resistant *Staphylococcus aureus* (MRSA) bacteremia, especially in patients undergoing hemodialysis, as it can be administered after dialysis [1,2]. However, daptomycin can lead to eosinophilic pneumonia, a rare but life-threatening side effect that typically develops two to four weeks after treatment initiation [3,4]. The exact incidence of daptomycin-related eosinophilic pneumonia remains uncertain. However, Soldevila-Boixader et al. reported that approximately 4.8% of a cohort of 229 patients developed this condition [5]. Several risk factors have been associated with its development, including age over 70 years, treatment duration longer than two weeks, higher cumulative doses of daptomycin, and an elevated Charlson Comorbidity Index. Additionally, patients who exhibit peripheral eosinophilia during daptomycin therapy appear to be at increased risk [4,5]. These factors should be carefully considered when managing patients receiving daptomycin to reduce the likelihood of DEP [5]. Daptomycin-induced eosinophilic pneumonia presents with fever, dyspnea, and pulmonary infiltration, often mimicking infectious pneumonia or septic emboli [6]. The clinical diagnosis of DEP is particularly difficult in patients undergoing hemodialysis, as these individuals exhibit overlapping symptoms resulting from volume overload, uremic pneumonitis, and frequent nosocomial infections [7]. In this report, we describe the case of a patient with persistent MRSA bacteremia undergoing hemodialysis who developed DEP. This case highlights the clinical importance of recognizing this specific adverse reaction and discontinuing daptomycin therapy to prevent severe respiratory failure and ensure clinical recovery.

## Case presentation

A 69-year-old man with a history of type 2 diabetes mellitus and end-stage renal disease secondary to diabetic nephropathy, undergoing maintenance hemodialysis, presented with altered mental status and fever. Clinical examination revealed septic shock (Table 1), swelling and tenderness over the right shoulder, and a purulent abscess on the sole of the right foot. MRSA was identified in blood cultures obtained from both peripheral and catheter sites. A transthoracic echocardiogram was performed and showed no vegetations. After removal of the dialysis catheter, intravenous vancomycin was initiated. Despite achieving therapeutic serum vancomycin levels, blood cultures remained persistently positive, likely due to the deep-seated nature of the infection within the joint and abscess. 

**Table 1:** Laboratory findings on admission WBC: white blood cell; eGFR: estimated glomerular filtration rate; CRP: C-reactive protein; INR: international normalized ratio.

Tests	Patient values	Reference ranges
Hemoglobin	112 g/L	130–170 g/L
WBC count	20.1 × 10⁹/L	4.0–11.0 × 10⁹/L
Neutrophil percentage	87.10%	40–70%
Eosinophil percentage	0.41%	1–4%
Platelet count	173 × 10⁹/L	150–450 × 10⁹/L
Blood urea nitrogen	15.6 mmol/L	2.5–7.1 mmol/L
Creatinine	608 µmol/L	60–110 µmol/L
eGFR	9 mL/min/1.73 m²	>60 mL/min/1.73 m²
Sodium	137 mmol/L	135–145 mmol/L
Potassium	5.5 mmol/L	3.5–5.0 mmol/L
Random blood glucose	12.9 mmol/L	3.9–7.8 mmol/L
Albumin	36 g/L	35–50 g/L
CRP	289.3 mg/L	<10 mg/L
Lactate	3.07 mmol/L	0.5–2.2 mmol/L
Blood cultures (peripheral and catheter)	Methicillin-resistant *Staphylococcus aureus*	Negative
Prothrombin time	13.0 s	10.0–14.0 s
INR	1.21	0.8–1.2

A metastatic infection workup identified septic arthritis of the right shoulder and a plantar foot abscess, both of which required surgical drainage. Because of persistent MRSA bacteremia, antimicrobial therapy was switched to daptomycin on hospital day 13 at a dose of 6 mg/kg administered after hemodialysis. Owing to persistent bacteremia, the dose was subsequently escalated to 9 mg/kg, consistent with evidence supporting high-dose daptomycin for refractory MRSA infection. Blood cultures showed clearance on hospital day 17.

**Figure 1:**
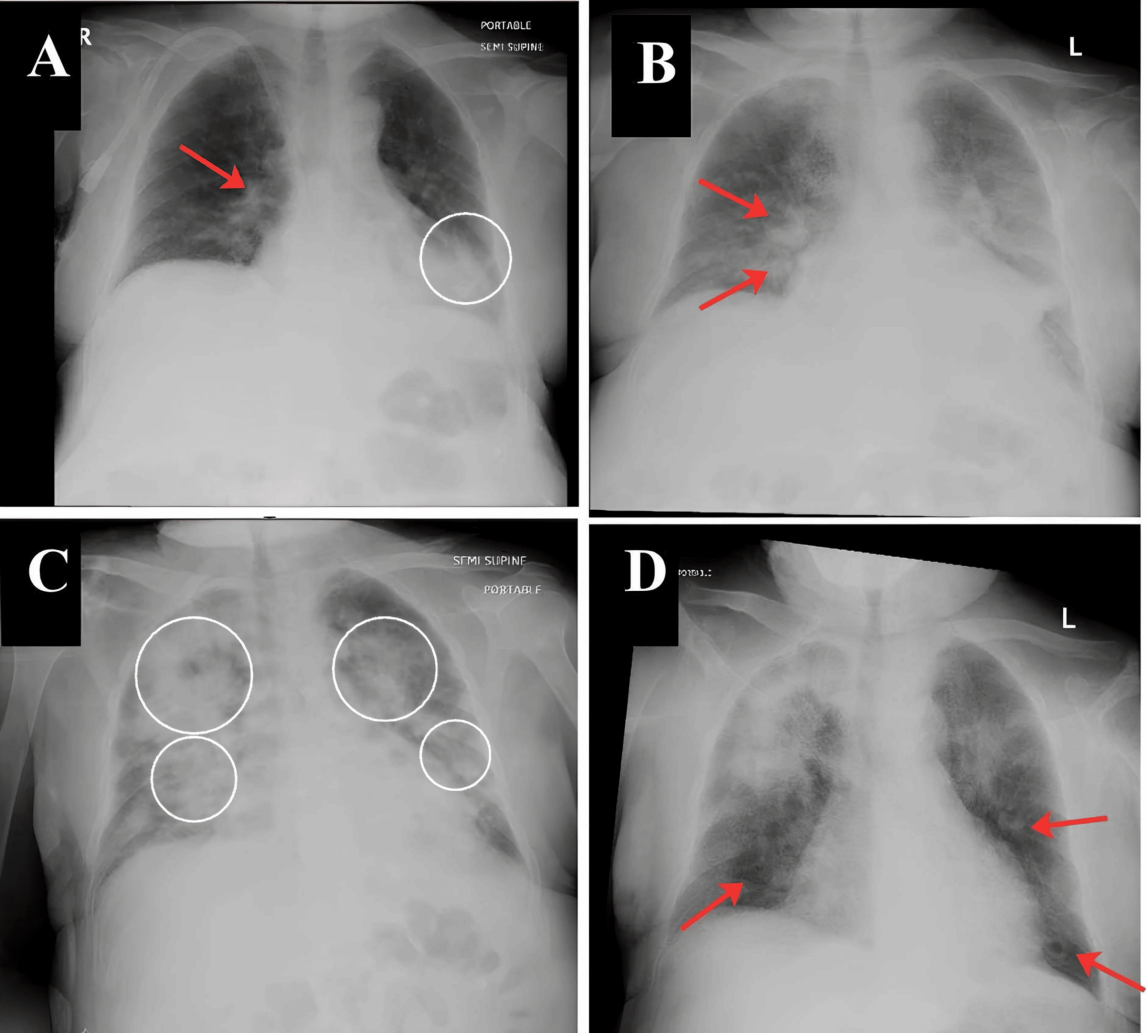
Serial portable chest radiographs show the progression and resolution of daptomycin-induced eosinophilic pneumonia (A) A baseline chest radiograph on admission (hospital day 0) shows prominent bronchovascular markings (red arrow) and left lower-zone atelectatic changes without focal consolidation (circle). (B) A chest radiograph on hospital day 37 (day 24 of daptomycin therapy) shows newly developed opacity in the right middle and lower lung zones (red arrows). (C) A chest radiograph on hospital day 40 shows progression to bilateral patchy air-space opacities (circles). (D) A chest radiograph on hospital day 43, 3 days after daptomycin discontinuation, shows improved aeration and near-complete resolution of infiltrates (red arrows).

**Figure 2:**
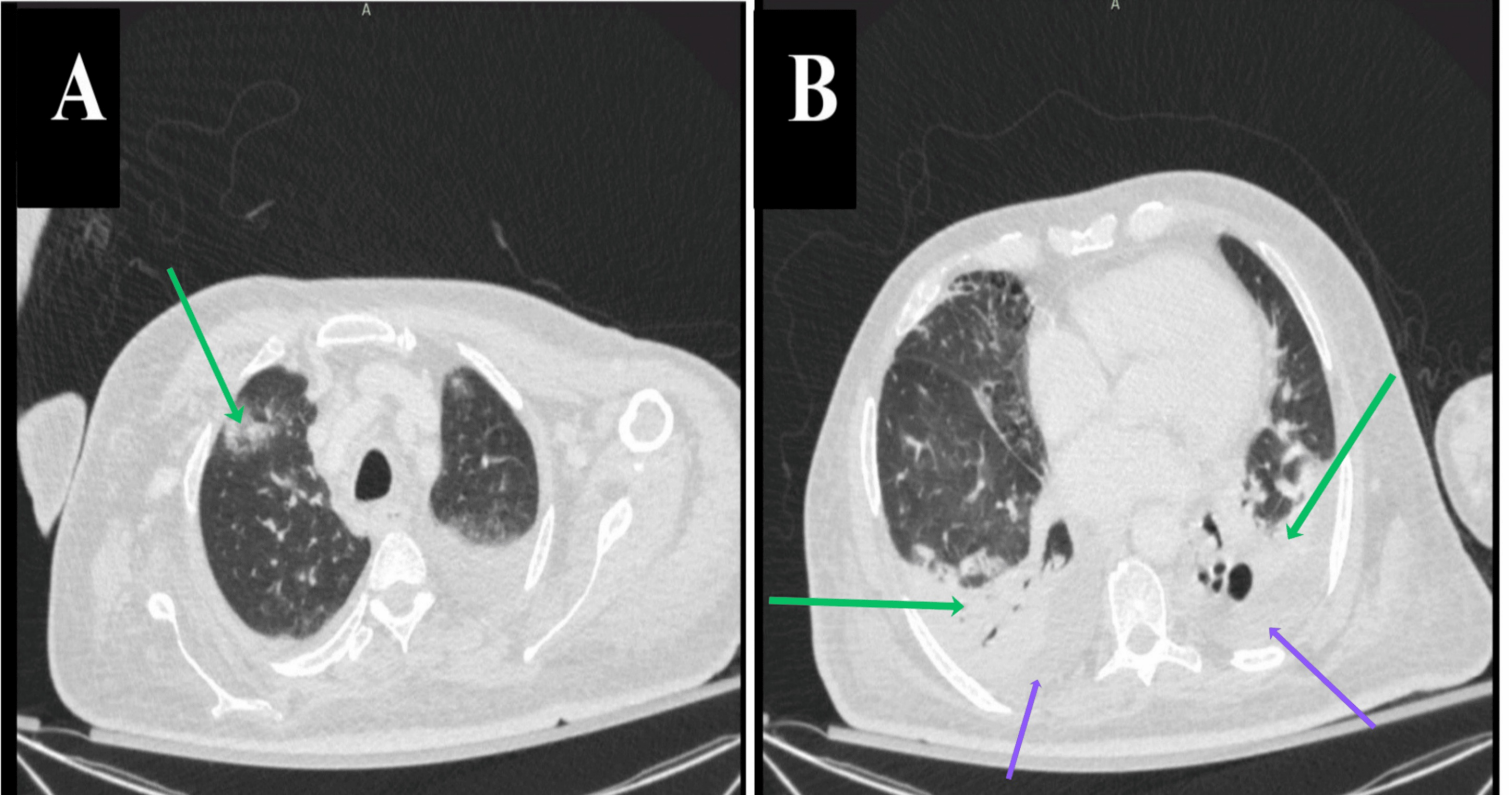
Axial computed tomography images of the chest obtained on hospital day 36 (day 23 of daptomycin therapy). (A) Scattered subsegmental areas of consolidation and ground-glass attenuation in the right upper lobe (green arrow). (B) Bilateral moderate pleural effusions (green arrows) with subsegmental consolidation of the basal segments of both lower lobes (purple arrows). The imaging demonstrates a predominantly peripheral and subpleural distribution of infiltrates and ground-glass opacities, a pattern more consistent with eosinophilic pneumonia than with the typical lobar consolidation of bacterial pneumonia or the wedge-shaped infarcts of septic emboli.

On day 24 of daptomycin therapy (hospital day 37), the patient developed acute hypoxemic respiratory failure, characterized by a fever (38.2°C), tachypnea (respiratory rate of 28 breaths/min), and progressive dyspnea. His oxygen saturation dropped to 85% on ambient air, requiring oxygen support via a high-flow nasal cannula at 40 L/min with an FiO2 of 0.60. An arterial blood gas analysis revealed severe hypoxemia (pH 7.42, PaCO2 38 mmHg, PaO2 58 mmHg). Chest radiography and computed tomography (CT) revealed new bilateral ground-glass opacities and patchy consolidations (Figures 1, 2), without evidence of septic emboli. Sputum cultures grew multidrug-resistant *Acinetobacter baumannii*. This was interpreted as colonization, as the patient was clinically euvolemic, showed no improvement with ultrafiltration, did not produce purulent sputum, and C-reactive protein levels did not significantly increase from the established baseline.

Concurrently, the peripheral eosinophil percentage increased from a baseline of 0.03% to a peak of 8.60% (Table 2). Although absolute eosinophil counts were not directly reported by the laboratory, calculation based on the total white blood cell count of 20.4 × 10⁹/L yields a peak absolute eosinophil count (AEC) of approximately 1.75 × 10⁹/L, well above the diagnostic threshold of 0.5 × 10⁹/L.

**Table 2:** Clinical timeline and major hospital events. WBC: white blood cell; AEC: absolute eosinophil count; CRP: C-reactive protein, MRSA: methicillin-resistant Staphylococcus aureus; CXR: chest radiography; —: Not performed or not applicable

Hospital day	Clinical event	WBC count (×10⁹/L)	Eosinophil %	Absolute Eosinophil Count (ACE)(×10⁹/L)	CRP (mg/L)
Day 0	Admission, MRSA identified, vancomycin initiated	20.1	0.41%	0.08	289.3
Day 12	Persistent MRSA bacteremia	23.6	1.57%	0.37	253.2
Day 13	Daptomycin initiated (6 mg/kg), surgical drainage of abscesses	26.9	1.42%	0.38	325.2
Day 15	Daptomycin dose increased (9 mg/kg)	—	—	—	—
Day 17	Bacteremia cleared (blood cultures negative)	—	—	—	—
Day 24	Clinical improvement, daptomycin continued	41.8	0.03%	0.01	187.8
Day 36	Eosinophils begin to rise (asymptomatic)	27.8	3.78%	1.05	190.0
Day 37	Respiratory distress onset: dyspnea, fever, new infiltrates on CXR	24.2	6.17%	1.49	224.7
Day 40	Peak eosinophilia, daptomycin discontinued, steroids initiated	20.4	8.60%	1.75	278.9
Day 41	Recovery: eosinophils normalized, hypoxia resolved	8.5	0.05%	<0.01	—

We suspected DEP based on the notable eosinophilia and its temporal correlation with daptomycin exposure. The patient exhibited several risk factors for DEP, including renal failure and prolonged administration of high-dose daptomycin. Other potential contributors, such as volume overload, septic pulmonary emboli, and bacterial superinfection, were considered less likely given the lack of response to ultrafiltration, negative echocardiogram, and clinical picture inconsistent with bacterial pneumonia. Daptomycin was discontinued on hospital day 40, and systemic corticosteroid therapy with methylprednisolone was initiated. Antimicrobial therapy was transitioned to ceftaroline for MRSA coverage. Within 24 hours of daptomycin cessation, the patient's oxygen requirements significantly decreased, and his eosinophil count normalized (AEC < 0.01 × 10⁹/L). Follow-up chest radiography indicated rapid improvement, with near-complete resolution of pulmonary infiltrates, supporting a probable diagnosis of DEP.

## Discussion

DEP is a rare but potentially fatal adverse drug reaction that has been increasingly recognized since the widespread use of daptomycin for invasive gram-positive infections [3,4]. Patients undergoing hemodialysis may be at increased risk due to altered pharmacokinetics and comorbidities. Common risk factors include older age, male sex, and high-dose daptomycin therapy [7]. Although the exact underlying mechanism remains unclear, DEP is thought to involve a T cell-mediated hypersensitivity reaction resulting in pulmonary eosinophilic infiltration [3,4]. In our case, potential contributors such as fluid overload and hospital-acquired pneumonia were carefully evaluated before a diagnosis of DEP. Although discontinuation of daptomycin is the primary intervention, corticosteroids were considered necessary in this patient to achieve rapid resolution of severe hypoxemia.

In the present case, the diagnosis of DEP was established based on its temporal relationship with daptomycin exposure, the development of new pulmonary infiltrates with hypoxemia, marked peripheral eosinophilia, and rapid clinical improvement following drug discontinuation, fulfilling the Solomon and Schwarz diagnostic criteria [8]. These criteria include: (1) exposure to daptomycin within the previous month; (2) new pulmonary infiltrates on imaging; (3) peripheral eosinophilia (AEC >0.5 × 10⁹/L or >10% of total white blood cells); and (4) exclusion of other causes of eosinophilic lung disease. Our patient met criteria 1, 2, 3 (with a peak AEC of 1.75 × 10⁹/L), and 4. The fifth criterion, bronchoalveolar lavage (BAL) with >25% eosinophils, could not be assessed due to the patient's clinical instability, a common limitation in such cases. The symptom onset on day 24 of therapy was consistent with the typical sensitization period of two to four weeks reported in the literature [3,9].

Diagnosing DEP in patients undergoing hemodialysis is particularly challenging because respiratory symptoms may be attributed to volume overload, uremic lung, or nosocomial infections. In our case, the presence of multidrug-resistant Acinetobacter baumannii in respiratory cultures represented a significant diagnostic confounder and could have led to inappropriate escalation of antimicrobial therapy. However, marked peripheral eosinophilia, which is uncommon in bacterial pneumonia, served as a key discriminating factor [10].

The rapid clinical improvement observed within 24 hours of daptomycin withdrawal and steroid initiation is noteworthy. While most DEP cases report improvement over 48-72 hours, the accelerated recovery in our patient may be attributable to the concurrent administration of systemic corticosteroids, which can potently suppress the underlying inflammatory response [3,4]. The decision to use corticosteroids in addition to drug withdrawal is often guided by the severity of respiratory failure. In patients with severe hypoxemia, as seen in this case, a combination approach is frequently employed to hasten recovery and mitigate the risk of further deterioration. However, this dual intervention makes it impossible to distinguish the independent effects of daptomycin cessation versus corticosteroid therapy on the patient's rapid recovery.

This study has several limitations. As a single case report, the findings may not be generalizable. The diagnosis of DEP remains probable rather than definitive because bronchoalveolar lavage, the gold standard for confirming pulmonary eosinophilia, was not performed due to the patient's clinical instability. This limits diagnostic certainty relative to established criteria. While evidence pointed toward colonization, the potential contribution of the concurrently isolated multidrug-resistant A. baumannii from respiratory cultures to the patient’s clinical picture cannot be entirely excluded. Finally, because corticosteroids were initiated at the same time daptomycin was withdrawn, it is not possible to determine the independent effects of either intervention on the patient's rapid clinical recovery.

## Conclusions

This case emphasizes the importance of maintaining a high index of suspicion for DEP in patients on hemodialysis who present with unexplained clinical deterioration of respiratory status during daptomycin therapy despite positive cultures for respiratory pathogens. Prompt diagnosis and discontinuation of the drug are necessary to prevent acute respiratory failure. Peripheral eosinophilia should alert clinicians to consider DEP and guide timely management.
